# Problem substance use in young adults: The impact of adverse life experiences

**DOI:** 10.1192/j.eurpsy.2025.580

**Published:** 2025-08-26

**Authors:** R. Fusco

**Affiliations:** Social Work, University of Georgia, Athens, United States

## Abstract

**Introduction:**

Data from community samples have shown that the risk of negative outcomes during the transition to adulthood is greater for those who engage in substance use. Alcohol use is common among young adults in the U.S., with 82% using in the past year and 34% engaging in havy episodic drinking . Marijuana is the second most widely used substance among young adults and regular use has been connected to an increased risk of anxiety and depression, and linked to both psychoses and poorer outcomes in people diagnosed with schizophrenia.

**Objectives:**

The goal of the current study is to understand the role of adverse childhood experiences (ACEs) on the likelihood of frequent marijuana and alcohol use among a sample of low-income young adults (*N*=182) in the U.S.

**Methods:**

Some original ACE study items were examined, such as types of child maltreatment. Additional items were added, including time spent in foster care as a child, involvement in a serious accident, experienced a serious injury, violent death of a family member or friend, and witnessed a serious injury or death. Frequent alcohol and marijuana use was defined as once a week or more in the past 30 days. Logistic regression models were developed to understand the magnitude of the contribution of each individual factor on the frequent use of both alcohol and marijuana. Separate models were developed to predict marijuana use and alcohol use.

**Results:**

The participants’ mean age was 20.5. The majority of the sample identified as Black (59%) or White (34%). A little more than half (53%) identified as female. Thirty percent reported using alcohol and 35% reported using marijuana at least once a week in the past 30 days.

The logistic regression model predicting frequent alcohol use showed several significant independent variables. Time in foster care (OR=2.88), childhood sexual abuse (OR=2.59), and childhood emotional abuse (OR=4.01) were all significant. Additionally, having a close family or friend who died violently (OR=6.63) and witnessing a serious injury or death (OR=2.92) were statistically significant.

The model predicting frequent marijuana use showed similar significant independent variables. Time in foster care (OR=3.48), childhood physical abuse (OR=3.67), childhood emotional abuse (OR=9.65), and having a close family member or friend who died violently (OR=4.99) all significantly increased the odds of marijuana use.

**Conclusions:**

Study results show the sustained negative impact of both child maltreatment and out-of-home placement. While less studied than other forms of maltreatment, emotional abuse was predictive of both alcohol and marijuana use. Youth who experienced violence in their community also showed a greater likelihood of alcohol and marijuana use. These results highlight the importance of identifying and addressing child maltreatment. Efforts to prevent and address substance use in young adults should take experiences of violence and abuse into account.

**Disclosure of Interest:**

None Declared

